# Supplementary material for: Deep sampling of Hawaiian Caenorhabditis elegans reveals high genetic diversity and admixture with global populations
Source: eLife. 2019 Dec 3;8:e50465. doi: 10.7554/eLife.50465 (PMC6927746; doi:10.7554/eLife.50465)
Supplement: Supplementary file 1. — Multiple collection categories were found for some samples. For this reason, the total number of distinct collections (2,594) exceeds the total number of samples (2,263). [file elife-50465-supp1.docx]

**Supplementary File 1**

| **Collection Category** | **Big Island** | **Kauai** | **Maui** | **Molokai** | **Oahu** | **Total** |
| --- | --- | --- | --- | --- | --- | --- |
| *C. elegans* | 31 | 1 | 5 | 1 | 0 | 38 |
| *C. oiwi* | 1 | 3 | 0 | 0 | 8 | 12 |
| *C. tropicalis* | 5 | 3 | 3 | 0 | 2 | 13 |
| *C. kamaaina* | 0 | 2 | 0 | 0 | 0 | 2 |
| *C. briggsae* | 20 | 49 | 22 | 0 | 4 | 95 |
| *Panagrolaimus* sp. | 7 | 1 | 3 | 1 | 0 | 12 |
| *Oscheius* sp. | 25 | 19 | 7 | 1 | 6 | 58 |
| *Teratorhabditis* sp. | 0 | 0 | 1 | 0 | 0 | 1 |
| *Rhabditis* sp. | 3 | 0 | 1 | 0 | 0 | 4 |
| *Choriorhabditis* sp. | 0 | 1 | 2 | 0 | 0 | 3 |
| *Mesorhabditis* sp. | 1 | 0 | 0 | 0 | 0 | 1 |
| *Chabertia* sp. | 1 | 0 | 0 | 0 | 0 | 1 |
| *Heterorhabditis* sp. | 1 | 0 | 0 | 0 | 0 | 1 |
| PCR - | 272 | 246 | 111 | 22 | 32 | 683 |
| Not genotyped | 150 | 207 | 123 | 19 | 28 | 527 |
| Tracks only | 193 | 89 | 116 | 23 | 10 | 431 |
| No Nematode | 124 | 407 | 129 | 23 | 29 | 712 |
| Total | 834 | 1028 | 523 | 90 | 119 | 2594 |
