# Supplementary material for: Deep sampling of Hawaiian Caenorhabditis elegans reveals high genetic diversity and admixture with global populations
Source: eLife. 2019 Dec 3;8:e50465. doi: 10.7554/eLife.50465 (PMC6927746; doi:10.7554/eLife.50465)
Supplement: Supplementary file 2. — The additional filters applied to the PopGen VCF for specific uses are described in methods. [file elife-50465-supp2.docx]

**Supplementary File 2**

| **VCF** | **Description** | **Use** | **Filter Parameters** | **Availability** | **Software used** |
| --- | --- | --- | --- | --- | --- |
| soft-filtered | Unfiltered variant set with soft-filters appended at variant and sample level | initial variant calling | Depth (DP) > 10;  Mapping Quality (MQ) > 40;  Variant quality (QUAL) > 10;  ((AD) / (DP)) ratio > 0.5; high_heterozygosity: >10% het calls ; high_missing > 90% sites missing | **CeNDR**  https://storage.googleapis.com/elegansvariation.org/releases/20180527/variation/WI.20180527.soft-filter.vcf.gz | BCFtools - variant calling and variant level filters; vcf-kit - append sample-level filters |
| hard-filtered | Any variant site flagged by the soft filters is removed, Any sample genotype with a soft-filter flag are set to missing | processed variant set | Same as soft-filter | **CeNDR**  https://storage.googleapis.com/elegansvariation.org/releases/20180527/variation/WI.20180527.hard-filter.vcf.gz | BCFtools - remove soft-filter sites |
| PopGen | Processed variant set for population genomics analyses | pi;  tajima's D;  Fst; phylogeny; neighbor-net;  admixture; treemix; haplotype | No missing genotypes sites | **Supplementary Data 3** https://github.com/AndersenLab/HawaiiMS/raw/master/data/elife_files/Supplemental_Data_3.vcf.gz | BCFtools - remove sites with missing genotypes |
