# Supplementary material for: Deep sampling of Hawaiian Caenorhabditis elegans reveals high genetic diversity and admixture with global populations
Source: eLife. 2019 Dec 3;8:e50465. doi: 10.7554/eLife.50465 (PMC6927746; doi:10.7554/eLife.50465)
Supplement: Supplementary file 3. [file elife-50465-supp3.docx]

**Supplementary File 3**

| **Field** | **Description** | **Type (units; if applicable)** | **Example values** |
| --- | --- | --- | --- |
| **C-label** | A label associated with a collection and obtained by scanning the barcode | Text | C-0001 |
| **Sample photo** | A photograph of the sample environment | Photograph | See figure 3C |
| **Substrate** | The sample substrate as determined in the field | Categorical | Leaf Litter, Fungus, orFlower |
| **Substrate notes** | Additional notes regarding a collected substrate | Text | Turned over log |
| **Landscape** | The type of environment from which a sample was obtained | Categorical | Wild forest Wild grassland |
| **Sky View** | The visibility of the sky from the perspective of the sample collected | Categorical | Full Partially Obstructed Obstructed |
| **Gridsect (optional)** | Whether the sample was part of a gridsect | Yes / No | Yes No |
| **Gridsect direction (optional)** | Only applies to gridset samples - Defines one of six directions collected within a gridsect | Categorical (degrees) | A, B, C, D, E, F |
| **Gridsect radius (optional)** | Only applies to gridsect samples - Defines the distance a sample was collected from the center | Categorical (meters) | 0 - Center  1  2  3 - Outer Circle |
| **Substrate temperature** | The temperature of the substrate as determined by local measurement | Numeric (ºC) | 16.4 |
| **Substrate moisture** | Moisture as determined by local measurement | Numeric (%) | 25 |
| **Ambient temperature** | The temperature of the environment | Numeric (ºC) | 18.2 |
| **Ambient humidity** | The humidity of the environment | Numeric (%) | 50 |
