# Supplementary material for: Deep sampling of Hawaiian Caenorhabditis elegans reveals high genetic diversity and admixture with global populations
Source: eLife. 2019 Dec 3;8:e50465. doi: 10.7554/eLife.50465 (PMC6927746; doi:10.7554/eLife.50465)
Supplement: Supplementary file 4. [file elife-50465-supp4.docx]

**Supplementary File 4**

| **Field** | **Description** | **Type** | **Example values** |
| --- | --- | --- | --- |
| **C-label** | The sample-collection bag | Text | C-0001 |
| **Worms on sample** | Whether nematodes were found associated with a sample | Yes / No | Yes |
| **Approximate number of worms (optional)** | An estimate of the number of nematodes found associated with a sample | Categorical | Very Few (1-3) Few (4-10) Some (11-25) Proliferating (25+) |
| **S-labeled Plates** | A list of S-labeled plates; Each S-label corresponds to a single nematode isolate | List | S-00001 S-00002 |
