# Supplementary material for: Deep sampling of Hawaiian Caenorhabditis elegans reveals high genetic diversity and admixture with global populations
Source: eLife. 2019 Dec 3;8:e50465. doi: 10.7554/eLife.50465 (PMC6927746; doi:10.7554/eLife.50465)
Supplement: Supplementary file 5. [file elife-50465-supp5.docx]

**Supplementary File 5**

| **Pipeline** | **Description** | **Availability** |
| --- | --- | --- |
| trimmomatic-nf | Performs trimming to remove poor quality sequences and technical sequences such as adapters. It should be used with high-coverage genomic DNA | <http://github.com/andersenlab/trimmomatic-nf> |
| alignment-nf | performs alignment for wild isolate sequence data on strain and isotype levels, and output BAMs and related information | <https://github.com/AndersenLab/alignment-nf> |
| concordance-nf | The concordance pipeline is used to detect sample swaps, identify samples with quality issues, and determine which wild isolate strains should be grouped together as an isotype | <https://github.com/AndersenLab/concordance-nf> |
| wi-nf | Calls variants for wild *C. elegans* isolates | <https://github.com/AndersenLab/wi-nf> |
